# Stress and Fatigue Management Using Balneotherapy in a Short-Time Randomized Controlled Trial

**DOI:** 10.1155/2016/9631684

**Published:** 2016-03-09

**Authors:** Lolita Rapolienė, Artūras Razbadauskas, Jonas Sąlyga, Arvydas Martinkėnas

**Affiliations:** ^1^Klaipeda Seamen's Health Care Center, Taikos Street 46, LT-91213 Klaipėda, Lithuania; ^2^Department of Nursing of Klaipeda University, H. Manto Street 84, LT-92294 Klaipėda, Lithuania; ^3^Klaipeda Seamen's Hospital, Liepojos Street 45, LT-92288 Klaipėda, Lithuania; ^4^Faculty of Health Science, Klaipėda University, H. Manto Street 84, LT-92294 Klaipėda, Lithuania; ^5^Department of Statistics, Klaipėda University, H. Manto Street 84, LT-92294 Klaipėda, Lithuania

## Abstract

*Objective*. To investigate the influence of high-salinity geothermal mineral water on stress and fatigue.* Method*. 180 seamen were randomized into three groups: geothermal (65), music (50), and control (65). The geothermal group was administered 108 g/L salinity geothermal water bath for 2 weeks five times a week. Primary outcome was effect on stress and fatigue. Secondary outcomes were the effect on cognitive function, mood, and pain.* Results*. The improvements after balneotherapy were a reduction in the number and intensity of stress-related symptoms, a reduction in pain and general, physical, and mental fatigue, and an improvement in stress-related symptoms management, mood, activation, motivation, and cognitive functions with effect size from 0.8 to 2.3. In the music therapy group, there were significant positive changes in the number of stress symptoms, intensity, mood, pain, and activity with the effect size of 0.4 to 1.1. The researchers did not observe any significant positive changes in the control group. The comparison between the groups showed that balneotherapy was superior to music therapy and no treatment group.* Conclusions*. Balneotherapy is beneficial for stress and fatigue reduction in comparison with music or no therapy group. Geothermal water baths have a potential as an efficient approach to diminish stress caused by working or living conditions.

## 1. Introduction

Stress is an important contributing factor to an individual's quality of life, and high levels of stress, if not managed, can negatively affect an individual's emotions, health, and implicit well-being [1–3]. Stress is linked to the six leading causes of death: heart disease, accidents, cancer, liver disease, lung ailments, and suicide [1]. It is also associated with an individual's absenteeism from work, increased medical expenses, loss of productivity, insomnia, fatigue, cognitive impairment, depression, and other mental or neurological illnesses, hypertension, arthritis, ulcers, asthma, migraines, immune system disturbances, skin diseases, aggression and relational conflict, and substance abuse and increases the negative effects of aging [3–6].

Stress can be caused by noise, vibrations, heat, improper lighting, and rapid acceleration in an individual's work pace, anxiety, fatigue, frustration, and anger [1, 2]. Stress is the second most frequently reported work-related health problem after musculoskeletal diseases, affecting 22% of workers in the European Union (EU) and accounting for 50–60% of all lost working days, with an annual cost of 20 billion Euro in 2002 (EU-15) [7]. Stress is widespread among health care and education workers and among other white-collar workers and seafarers [7–10]. Seafaring is a dangerous and challenging profession worldwide and is associated with a high level of work-related stress [11–13]. In the EU, prevention of work-related stress is one of the most important new strategies for health and safety at work [14].

Stress has a direct association with fatigue and health [2]. The prevalence of fatigue in the general working population has been estimated to be as high as 22%, and this fatigue is associated with pain, tiredness, nervous system mechanisms, and environmental stimuli in individuals who experience the effects [1, 15]. The effects of fatigue are multifaceted and complex, overlapping various biological areas such as performance, physiology, cognition, and emotion [1].

Health promotion and disease prevention are more important than ever in societies with increasing life expectancy and growing stress levels. Health promotion measures can help prevent the onset of a disease and may have a beneficial effect on the course of the disease, thus contributing to healthy aging with good quality of life [16]. Numerous approaches are available for stress and fatigue management that can decrease patients' suffering from stress and fatigue and enhance their quality of life, but no single approach fits for all individuals. Stress model demonstrates the favorable effects exhibited by sleep, recovery, and social support [2]. It has been proven that relaxation techniques such as behavioral therapy, meditation, yoga, breathing techniques, reflexology, massage, Reiki, water therapy, and others are beneficial for stress reduction [11, 17–21]. Specifically, a technique called music therapy involves the use of music therapeutically to address physical, psychological, cognitive, and/or social functioning for patients of all ages. According to researchers, music therapy improves human psychological conditions, reduces symptoms of anxiousness and depression, and treats long-term stress-related physical ailments [12, 17, 19].

Balneotherapy (lat. balneum – bath + gr. therapeia – treatment, nursing) involves using natural mineral spring water for the prevention and cure of disease. The World Health Organization recognizes the therapeutic impact of medicinal mineral waters, thermal or nonthermal [20]. Natural mineral water is a general term applied to both spring and other underground continental waters (from deep-seated water-wells). Because of high temperature and mineralization, geothermal water can serve people from various economical areas for improving health during balneotherapy procedures [13, 22]. Natural mineral water has been considered a curative tool for millennia, having well-known healing properties in terms of prevention, treatment, and rehabilitation of skin, musculoskeletal, cardiovascular, endocrine, nervous, and other human body systems [11, 16, 20, 23–28]. During the last 30 years, a number of controlled trials have demonstrated the efficacy of balneotherapy in treating certain diseases, mostly musculoskeletal conditions [16, 25, 27, 29].

The essence of balneotherapy effects (including antistress) are local changes caused by the direct influence of mechanical, thermal, and chemical factors through the skin and mucous membranes and complex adjustment reactions as a result of neuroreflexive, humoral mechanisms, caused by stimulation of mechano-, thermo-, baro-, and chemoreceptors by biochemical active substances during the balneoprocedure [21, 30–36].

Researchers in balneotherapy studies commonly used mineral water of 0.6–31.9 g/L total mineralization, excluding studies in the Dead Sea [22, 23, 25, 28, 33–35, 37–39]. We performed the study with 108 g/L TDS in order to increase the effectiveness for the general working population. To our knowledge, there have not been any trials with such a high salinity of thermal water. This study may be the first published in English applying such a highly concentrated brine application in which the mineral age of the water is not less than 1 million years.

*The objective* of our study was to investigate the influence of high-salinity geothermal mineral water from artificial sources on seamen's stress and fatigue and to find an effective measure of health protection and restoration for individuals working in highly stressful occupations.

## 2. Patients and Methods

This prospective open-label randomized controlled parallel-group biomedical trial was implemented in observance of the rules of good clinical practice; our research protocol was approved by the Kaunas Regional Biomedical Research Ethics Committee (Approval number BE-2-31/2012). All subjects were informed about the purpose, conditions, and course of the study prior to the inclusion and signed a participant's agreement.

This study was conducted in Klaipėda, Lithuania, during September–November, 2012. The target population consisted of working seamen from Klaipėda region (Lithuania), recruited and examined by a trained independent general practitioner (GP) during a medical examination at a Sea Center in the Seamen's Hospital and Seamen's Health Care Center in Klaipėda city. Out of the 220 seamen, 180 met inclusion criteria and agreed to participate in the study and were subject to a randomization procedure. Inclusion criteria were as follows: male seamen, aged 25–64, and working at sea for more than 5 years, stress and fatigue intensity level more than 2 (visual analogue scale (VAS) from 0 to 10). Our exclusion criteria were related to the following symptoms: acute organic neurological deficit, neoplastic or inflammatory lesion, decompensated cardiovascular disease, unstable metabolic disorders, febrile infections, and cutaneous suppuration.

**Figure 1:**
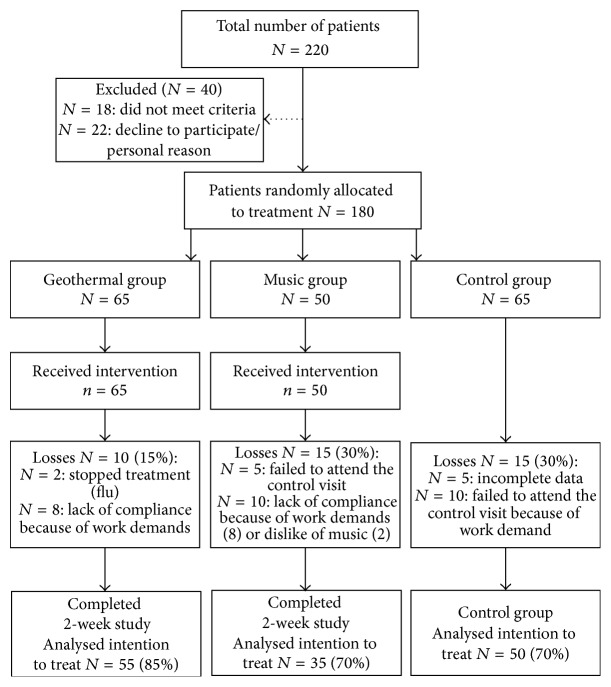
Disposition of the study participants.

After the completion of survey for sociodemographic, work-related, and clinical data, 180 subjects were randomized into three groups: the balneotherapy group (65), the music therapy group (50), and the control group (65). The disposition of the participants is shown in Figure 1. The randomization was simple, with generation of random numbers. The generation of random number order was performed by a professional using the SPSS v. 21 software package. The independent professional performing the statistical analysis was aware of the randomization. The enrolled patients completed the balneotherapy and music therapy treatments as outpatients, with no change in their daily routine or work attendance. The same instructions were made for the participants of the control group. The side effects of balneotherapy were supposed to be evaluated by the physician supervising the treatments by means of an observational sheet, side effects of music therapy, in self-administered observational sheets.

The numbers of participants included in the analysis were as follows: in the geothermal group, 55 subjects were analyzed in order to determine the influence of the therapy on stress and fatigue, as well as its effect on the cardiopulmonary system, mood, and pain; in the music therapy group, data from 35 subjects were studied, and, in the control group, 50 participants were analyzed.

The balneotherapy group was administered a head-out immersion bath with naturally warm (34.6°C on average) highly mineralized (108 g/L) geothermal Na-Cl-Ca-Mg-SO_4_ mineral water with a pH 6.07 from a “Geoterma2P” borehole (1135 m depth, Lower Devonian layer, mineral age of more than 1 million years). Volumetric activity of radon in the water was 29 ± 5 Bq/L. The water chemical composition is shown in Table 1.

The subjects underwent balneotherapy sessions for 15 min daily, five times a week, for 2 weeks, and were monitored continuously during the treatment sessions by trained personnel. Each participant was told to move slightly in the bathtub during the procedure. After the baths, participants were recommended to gently dry the skin with a towel and not to shower for about one hour to prolong the effects of the procedure [20]. The study protocol required that participants attend at least 60% of treatments or a minimum of 6 balneotherapy sessions. We performed ten procedures of music therapy at home with Peter Huebner's Medical Resonance Therapy Music^©^ (RRR 932 General Stress) [40]. We used the receptive intervention of music therapy, which asks participants to listen to music, allowing them to become recipients of the musical experience. We used standardized music therapy procedures approved by the Rehabilitation Department of Klaipėda Seamen's Hospital and recommended by Medical Resonance Therapy Music, which involve treatment through the following process: 20 minutes of sitting or lying down with closed eyes and earphones and avoiding disturbances from outside noise. The participants were trained for the music therapy procedure and for blood pressure and heart rate measurement. They were given self-observational protocols and were asked to record any changes in their general feelings after the procedure. Adherence to the self-administered music therapy protocol was controlled by daily telephone calls.

Baseline and posttherapy assessments of all three groups were performed by a trained GP. The GP's evaluation consisted of overall reported health, medication use, evaluation of pain using visual analogue scale (VAS) from 0 to 10 (0 = no pain; 10 = worst imaginable pain), mood according to Likert's 5-point scale ((1) bad, (2) satisfactory, (3) good, (4) very good, and (5) perfect mood), systolic and diastolic blood pressure (mmHg), and heart and respiratory rates (time/min) as changes in feelings and side effects. Stress and fatigue were assessed by the self-administered general symptoms distress scale (GSDS) [41], the Multidimensional Fatigue Inventory (MFI) [42], and the Cognitive Failures Questionnaire (CFQ) [43].

The primary outcome measures were as follows. (1) First is the change of distress level in the GSDS between baseline and posttreatment evaluations. The GSDS (T. Badger, Arizona, USA) was chosen due to its adequate internal consistency, reliability, good constructional and prognostic validity, and good correlation with depression and positive and negative affects [41]. This short psychometric tool allows for assessing specific distress symptoms and evaluating their intensity and control on a 10-point scale. (2) Second is the changes in fatigue scores (MFI). The MFI is a 20-item self-report instrument designed to measure fatigue. It covers the following dimensions: general fatigue, physical fatigue, mental fatigue, reduced motivation, and reduced activity. The use of this instrument offers the opportunity to obtain a profile of fatigue [42]. The secondary outcome measures of the study included such self-assessment scales as CFQ, mood and pain scales, changes in the cardiopulmonary system (blood pressure, heart rate, and respiration rate), and medication use.

The sample size was estimated using the IBM SPSS Sample Power Release software v. 3 for the stress outcome using the general symptoms distress scale (GSDS). We examined mean differences between balneotherapy and the control groups. We estimated that the sample size in both groups should be 32 subjects, with the power of 81.7% to achieve a statistically significantly different result. This computation assumes that the mean difference in the general symptoms distress between the balneotherapy and the control or the music therapy groups would be not less than 0.8, and the standard deviation within the groups would be 1.1. This effect was selected as the least significant effect of detectable importance; any smaller effect would not be of clinical or substantive significance. We assumed that the influence of balneotherapy and music therapy on the difference in the mean values of the variables is valid because such changes during the procedures are fully probable in this field of research. The mean difference of the observed variables of 0.8 (1.1) would be presented with a 95% CI of 0.25 to 1.35.

## 3. Statistical Analysis

A descriptive analysis (mean, standard deviation (SD), frequencies, and percentages) was used to examine the participants' background, demographic variables, and treatments. Data were presented with mean (SD). Data distribution was assessed by applying the Kolmogorov-Smirnov test.

We used the parametric criteria and Student's *t*-test to determine mean differences between two groups; the ANOVA with Bonferroni difference test was applied for multiple comparisons if equal variances were assumed, and Tamhane's *T* ^2^ multiple comparison test was used if equal variances were not assumed between the three groups. Due to the nonnormality of the distribution of the variables, nonparametric methods (the Mann-Whitney test) were used in statistical analyses. The categorical variables between the groups were compared using the chi-squared (*χ* ^2^) and Fisher's exact test. *p*-values less than 0.05 were interpreted as statistically significant.

Paired effect sizes before and after the treatment in the groups were estimated with a 95% confidence interval (CI).

The results were evaluated by applying the intention-to-treat analysis (ITT).

The data were analyzed using SPSS (version 21.0; SPSS Inc., Chicago, IL, USA) software.

## 4. Results

The subjects' sociodemographic and clinical characteristics and health-related issues are shown in Table 2. All three groups were similar concerning sociodemographic characteristic, working conditions, stress, fatigue, and pain frequency and intensity, the perceived health status, and harmful addictions. Lower body mass indices and lower medication usage, yet higher morbidity, were found in the geothermal group. All groups were similar concerning neurological diseases, and a significantly bigger morbidity in musculoskeletal, gastrointestinal, and urological and ear, nose, and throat (ENT) diseases was seen in the geothermal group (Table 2).

After a 2-week treatment, patients receiving geothermal water therapy showed a significant therapeutic response compared to the control and music therapy groups (Table 3).

The inside-the-group analysis showed significant positive changes in primary and secondary (CFQ, mood, and pain) outcome measures with the large effect size from 0.78 to 2.25 (*p* < 0.001). The biggest effect was observed for decreasing stress symptoms, pain, stress intensity, and general fatigue (Table 3).

A significant positive effect of music therapy was seen in the reduction of stress symptoms (large effect) and the intensity of stress (medium effect) and pain (medium effect), as well as an increase in activity (small effect) and mood (medium effect).

During the inside-the-group analysis, the participants of the control group showed significant negative changes in health status concerning stress management, all fatigue dimensions, and cognitive function. No positive changes were seen after 2 weeks (Table 3).

Group comparison is as follows. The changes in the intervention groups (G versus M) were significant in lowering the number of stress symptoms (medium effect size 0.5, 95% CI −0.9 to −0.05 (bias corrected by Hedges)), decreasing pain intensity (large effect size 0.95, 95% CI −1.4 to −0.5), and improving of mood (medium effect size 0.6, 95% CI 0.17 to 1.03) (Table 3). No significant differences were found in other stress items as well as some fatigue dimensions between groups because of significant differences in baseline data.

The comparison between the geothermal and the control groups showed a significant positive therapeutic effect in geothermal group on all primary (stress and fatigue) and secondary (mood and pain) outcomes despite certain baseline clinical differences between the groups, which were unfavorable to the geothermal group (Tables 2 and 3). Large effect sizes between G versus C groups were found in lowering the stress symptoms (1.2, 95% CI −1.65 to −0.81), pain (1.05, 95% CI −1.46 to −0.65), and general fatigue (1.06, 95% CI −1.47 to −0.65) and in increasing stress management (0.8, 95% CI 0.42 to 1.21), activity (−0.89, 95% CI −1.29 to −0.48), and mood (1.16, 95% CI 0.74 to 1.57); a medium effect size was seen in reducing physical (0.73, 95% CI 1.13 to −0.34) and mental fatigue (0.53, 95% CI −0.92 to −0.14) and increasing motivation (0.65, 95% CI −1.04 to −0.25); and a small effect size was observed for reducing stress intensity (0.38, 95% CI −0.77 to 0.01).

After 2 weeks of balneotherapy treatment, changes in other study secondary outcomes—cardiopulmonary system parameters—were seen: a reduction of systolic (from 136.4 to 129.5 mmHg; mean difference, 7 mmHg; CI 3.03 to 11.7; *p* = 0.001) and diastolic (from 84.3 to 78 mmHg; mean difference, 6 mmHg; CI 3.22 to 8.06; *p* < 0.001) blood pressure, heart rate (from 87.3 to 72.4 bpm; mean difference, 15 bpm; CI 1.03 to 5.0; *p* = 0.004), and respiratory rate (from 15.7 to 14.3 times/min; mean difference, 1.4 times/min; CI 0.47 to 1.7; *p* = 0.001), while in the music therapy group, only the respiratory rate was significantly reduced (from 15.6 to 14.7 times/min; mean difference, 0.9 times/min; CI 0.23 to 1.49; *p* = 0.009). No significant changes were seen in the control group. The differences between the groups were significant (*p* < 0.001).

In the geothermal therapy group, less medication use was observed (*p* = 0.047, *z* = 2.0); no significant changes were observed in the music therapy or the control groups.

Adverse events during balneotherapy were assessed and registered by a GP; they were mild and transient: skin irritation (redness, rash), 4.6% (3), and exacerbation of psoriasis, 1.5% (1). However, no patients had to discontinue treatments because of adverse events. The side effects of music therapy included headaches (3%) (1) and annoyance due to the dislike of the music (3%) (1).

## 5. Discussion

The results of the study demonstrated that the 2-week geothermal bath had a positive effect on stress, fatigue, mood, pain, and cognitive function, as cardiopulmonary function. Compared to music therapy, balneotherapy showed a more significant effect in reducing stress symptoms and pain and in improving the mood of the participants. When counting large-medium effect sizes, geothermal water treatment was more effective in relieving stress- and fatigue-related symptoms and pain, compared to the results of the control groups. Our study proved the safety of geothermal very high-salinity water as a treatment for the general working population.

A number of trials exploring the benefits of balneotherapy to humans have been conducted [16, 20, 23, 25, 27]. The combined effects of balneotherapy have been shown to result in positive outcomes related to rheumatic, skin, cardiovascular, pulmonary, endocrine, and mental diseases and in the improvement of public health [22–29, 37–39, 44–47]. However, spa medicine is not yet sufficiently recognized in psychiatry [23]. The effect of relaxation, a sense of well-being, and a reduction of stress are associated with changes in hormones like cortisol or endogenous opiates [21, 35]. Our trial results correlate with positive effects of balneotherapy related to relieving stress, pain, feelings of depression, and burnout and improving the quality of life, sleep, psychoemotional well-being, and mental activity [18, 25, 37, 45–49]. Dubois et al. provided the first research-based proof that balneotherapy was effective and well-suited for the treatment of generalized anxiety disorder. The mean change in Hamilton's total score after 8 weeks was significantly greater in the balneotherapy than in the paroxetine group (−12 versus −8.7; *p* < 0.001); the change in the Montgomery-Asberg Depression Rating Scale was bigger in the balneotherapy group (−8.4 versus −7; *p* = 0.04) [44]. The 3-week trial with Lintong mineral spring water demonstrated a positive effect on tension (from 4.75 to 2.17), anger (from 2.09 to 0.88), fatigue (from 3.46 to 1.12), confusion (from 3.36 to 1.17), and vigor (from 15.87 to 21.71) mood scores (*p* < 0.05), and the mood state of depression-dejection also showed a decreasing trend (from 0.5 to 0.36) [37]. Positive results of a 3-week spa therapy were also observed by Blasche in patients with breast cancer (improved mood and the quality of life) and occupational burnout (reduced general fatigue, distress, and increased motivation) [46]. Compared to resting and progressive muscle relaxation, balneotherapy was more beneficial with regard to subjective effects of relaxation and similarly effective with regard to a decrease in salivary cortisol levels [48]. According to the results of a study by Latorre-Román et al., 12-day balneotherapy had a positive effect on pain, mood, sleep quality, and depression in healthy older people: pain decreased by 1.2 on VAS (*p* = 0.001), depression by 0.18 (*p* = 0.03), anxiety by 0.38 (*p* = 0.001), tension by 0.37 (*p* = 0.001), and fatigue by 0.35 (*p* = 0.001), whereas vigor increased by 0.18 (*p* = 0.049) [49].

There is a strong association between stress and pain [2, 10], and the analgesic effect of thermal water is well-known and confirmed in meta-analyses and systematic reviews [23–25, 27, 50]. Naumann and Sadaghiani conducted a systematic review and meta-analysis of randomized trials on hydrotherapy and balneotherapy in fibromyalgia patients, which showed moderate evidence for a significant reduction of pain at the end of the balneotherapy treatment (standard mean difference (SMD) −0.84; *p* = 0.002) and for a medium improvement in the quality of life (SMD −0.78; *p* < 0.0001); there was no significant effect on depressive symptoms (SMD −0.87; *p* = 0.07) [50]. All the reviewed studies on lower back pain reported that balneotherapy was superior in long term to tap water therapy in relieving pain and improving function and that spa therapy combining balneotherapy with mud pack therapy and/or exercise therapy, physiotherapy, and/or education was effective in the management of low back pain and superior or equally effective to the control treatments in short and long terms [51]. The various articles are exploring the importance and significance of traditional/alternative way of pain treatment with conclusion that pain is best controlled using coordinated efforts of both traditional and alternate measures [52]. Our trial demonstrated that after 2 weeks of procedures, systolic (by 7 mmHg) and diastolic (by 6 mmHg) blood pressure, as well as heart rate (15 bpm) and respiratory rate (1 time/min) lowered among our subjects. This finding is in line with the results of a study conducted by Xu et al., demonstrating improved cardiopulmonary function after thermal baths [38] and safety of the therapy for participants with mild to moderate hypertension [33, 34, 39, 47]. Becker et al. have revealed hydrotherapy effects on lowering blood pressure, 12 mmHg in systolic blood pressure and 26 mmHg in diastolic blood pressure [31]; other scientists found that one mineral bath is lowering systolic blood pressure 2–15 mmHg and reducing heart rate 5-6 t/min [53].

The ability to rest, relax, and recuperate is a form of self-treatment and an important aspect in combating the harmful effects of cumulative stress [17]. Since each person responds to stress differently, there is no single effective stress reduction strategy. Individuals may successfully achieve work- or life-related stress reduction through multidisciplinary work utilizing an integrated approach [7, 14]: organizational risk prevention [5, 8], changing one's mindset from “stress-is-debilitating” to “stress-is-enhancing” (which can profoundly influence psychological, behavioral, and physiological outcomes) [17], living a healthy lifestyle, and using interventions to balance or minimize stress intensity [54] with the help of population-based wellness strategies. Balneotherapy using geothermal water may be a valuable strategy to use [23, 29, 46, 48, 49].

To our knowledge, this study is the first to explore the effect and safety of geothermal mineral water therapy with very high-salinity water. Our results showing big promises for relieving psychoneurological conditions in humans might be related to water mineralization or its origin. The limitations of our study are significant differences in certain scale dimensions between the groups that emerged during the initial stages of the study, the evaluation of the effect of the therapy on the representatives of a single profession and by a single GP, subjective measures of stress and fatigue, administration of music therapy at home, open-label trial, and no follow-up. The challenge for the future will be to carry out well designed studies in larger patient population with different water mineralization levels and follow-up. The results of our study results demonstrate a need for further research in balneotherapy using geothermal waters as a stress-reducing tool as well as a separate tool for disease prevention and treatment.

## 6. Conclusions

1. Geothermal water baths reduce stress, fatigue, and pain, improve mood, stress management and cognitive functions, and have a positive effect on the cardiopulmonary system.
2. Balneotherapy using geothermal water proved to be more efficient for relieving stress and pain than music therapy was, whereas, for all stress- and fatigue-related conditions, it was more efficient than no therapy.
3. Balneotherapy could be an effective measure for stress prevention and health restoration as an integral part of a multimodal work-related stress-reducing program.

## Figures and Tables

**Table 1:** The mineral composition of geothermal water.

Element	Concentration, mg/L
Cl^−^	66930
Na^+^	27580
Ca^2+^	8990
Mg^2+^	2630
SO_4_^2−^	1330
K^+^	690
HCO_3_^−^	74
Br	60.62
N	22
Fe	12.14
B	6.501
Si^4+^	4.886
Li^+^	1.2
Cr	1
F^−^	0.91
Mn^2+^	0.501
H_2_S	0.33
Cu^2+^	0.167
Zn^2+^	0.062
Total amount of dissolved mineral substances (TDS), mg/L	**108334**

**Table 2:** Sociodemographic and clinical characteristic of the participants in groups.

	Geothermal (*n* = 55)	Music group (*n* = 35)	Control (*n* = 50)	*p* value
Age, years, mean (SD)^a^	47.5 (10.6)	47.6 (10.7)	46.2 (9.3)	0.733
Education, *N* (%)^b^				
Secondary school	10 (19.2)	4 (11.8)	4 (8.3)	0.304
College	26 (41.9)	10 (29.4)	21 (43.8)	
Higher education	20 (32.3)	18 (52.9)	21 (43.8)	
Marital status, *N* (%)^b^				
Single	8 (15.1)	4 (11.4)	2 (4.1)	0.590
Married	36 (67.9)	27 (77.1)	40 (81.6)	
Work experience, years, mean (SD)^a^	22.5 (11.4)	23.1 (11.5)	22.4 (9.9)	0.950
Working hours per day, *N* (%)^b^				
8–12 hours	32 (58.2)	19 (54.3)	34 (68.0)	0.302
13–15 hours	21 (38.2)	11 (31.4)	12 (24.0)	
16–18 hours	1 (1.8)	3 (8.6)	3 (6.0)	
>18 hours	1 (1.8)	2 (5.7)	0	
Resting hours in 24 hours, *N* (%)^b^				
10 hours	12 (21.8)	3 (8.6)	8 (16)	0.366
8 hours	17 (30.9)	15 (42.9)	12 (24)	
6 hours	20 (36.4)	14 (40.0)	21 (42)	
<6 hours	6 (10.9)	3 (8.6)	9 (18)	
Leading work position, *N* (%)^b^	19 (35.2)	14 (46.7)	21 (43.8)	0.433
Frequent stress, *N* (%)^b^	15 (27.3)	10 (28.6)	14 (28.0)	0.405
Frequent fatigue, *N* (%)^b^	20 (36.4)	9 (25.7)	10 (20.8)	0.149
Frequent pain, *N* (%)^b^	4 (7.3)	1 (2.9)	4 (8.0)	0.100
Stress intensity, VAS, cm, mean (SD)^a^	3.8 (1.6)	3.7 (1.9)	3.6 (1.7)	0.820
Pain intensity, VAS, cm, mean (SD)^a^	3.1 (1.7)	2.9 (1.7)	2.4 (1.6)	0.131
Fatigue intensity (0–7), cm, mean (SD)^a^	3.4 (1.3)	3.3 (1.1)	3.3 (1.0)	0.746
Insufficient sleep, *N* (%)^b^	17 (30.9)	8 (22.9)	12 (24.5)	0.848
BMI, mean (SD)^a^	27.1 (3.0)	28.9 (3.0)	26.7 (5.1)	0.040
Morbidity, *N* (%)^b^	51 (94.4)^c^	28 (80.0)^d^	34 (68.0)^d^	0.002
Cardiovascular diseases	28 (50.9)	12 (34.4)	16 (32.0)	0.103
Musculoskeletal diseases	48 (87.3)^c^	19 (54.3)^d^	29 (58.0)^d^	0.001
Gastrointestinal diseases	24 (43.6)^c^	11 (31.4)^c^	6 (12.0)^d^	0.002
Neurological diseases	27 (49.1)	14 (40.0)	20 (40.0)	0.571
Respiratory diseases	9 (16.4)	4 (11.4)	4 (8.0)	0.419
ENT diseases	10 (18.2)^c^	1 (2.9)^c^	3 (6.0)^d^	0.031
Urological diseases	19 (34.5)^c^	2 (5.7)^d^	6 (12.0)^d^	0.001
Medication usage, *N* (%)^b^	19 (29.7)	20 (57.1)	23 (46.9)	0.021
Amount of cigarettes per day, units, mean (SD)^a^	13.5 (6.2)	10.4 (6.3)	14.1 (9.3)	0.399
Amount of alcohol units^*∗*^ per week, mean (SD)^a^	3.75 (3.1)	4.45 (5.84)	3.25 (2.35)	0.384
Good health state, *N* (%)^b^	32 (58.1)	20 (57.1)	30 (60)	0.332

^a^ANOVA test with Bonferroni correction, ^b^chi-squared (*χ* ^2^) test, and ^c,d^ *z*-test, between proportions from each other *p* < 0.05.

^*∗*^1 unit = 0.5 pint of beer/lager or a small glass of wine or a pub measure of spirits or a small glass of sherry or port.

**Table 3:** The effect of changes stress, fatigue, and pain, follow-up compared with baseline; comparison of changes and of treatment groups.

Item	Geothermal, *n* = 55				Music group, *n* = 35				Control group, *n* = 50				Group comparison	
	Baseline	Changes compared to the baseline			Baseline	Changes compared to the baseline			Baseline	Changes compared to the baseline			G-M	G-C
	After				After				After				*p*	*p*
	Mean (SD)	Mean 95% CI	Effect size^a^ 95% CI	*p* value	Mean (SD)	Mean 95% CI	Effect size^a^ 95% CI	*p* value	Mean (SD)	Mean 95% CI	Effect size^a^ 95% CI	*p* value	B A	B A
Stress symptoms	4.35 (1.85)	−2.64	2.25	<0.001	3.09 (1.58)	−0.71	1.13	<0.001	3.32 (1.77)	0.06	−0.05	0.722	0.003	0.010
	1.71 (1.38)	−2.98 to −2.30	1.94 to 2.55		2.37 (1.37)	−0.94 to −0.49	0.79 to 1.48		3.38 (1.31)	−0.28 to 0.40	−0.36 to 0.25		0.029	<0.001

Stress intensity	5.41 (1.78)	−2.25	1.31	<0.001	3.54 (1.72)	−0.54	0.70	<0.001	3.82 (1.83)	−0.02	0.02	0.894	<0.001	<0.001
	3.16 (1.95)	−2.71 to −1.78	0.96 to 1.66		3.00 (1.63)	−0.81 to −0.28	0.30 to 1.09		3.80 (1.29)	−0.32 to 0.28	−0.29 to 0.33		0.681	0.050

Stress management	5.64 (1.99)	1.98	−0.82	<0.001	6.77 (2.57)	0.40	−0.22	0.221	6.44 (2.05)	−0.44	0.32	0.033	0.050	0.179
	7.62 (2.21)	1.33 to 2.64	−1.21 to −0.43		7.17 (2.16)	−0.25 to 1.05	−0.77 to 0.34		6.00 (1.68)	−0.84 to 0.04	−0.05 to 0.69		0.348	<0.001

General fatigue	46.36 (26.34)	−23.75	1.22	<0.001	34.64 (22.86)	−3.21	0.24	0.168	35.38 (19.75)	5.1	−0.63	<0.001	0.064	0.051
	22.61 (17.45)	−29.43 to −18.07	−2.95 to 5.40		31.43 (25.79)	−7.85 to 1.42	−5.47 to 5.95		40.50 (15.94)	2.60 to 7.65	−4.15 to 2.89		0.056	<0.001

Physical fatigue	37.62 (25.54)	−16.78	0.88	<0.001	34.29 (21.30)	−4.64	0.24	0.175	27.63 (17.99)	5.88	−0.87	<0.001	1.000	0.053
	20.83 (18.85)	−22.25 to −11.32	−3.32 to 5.07		29.64 (24.92)	−11.46 to 2.17	−5.19 to 5.67		33.50 (15.14)	3.80 to 7.95	−4.13 to 2.39		0.061	<0.001

Reduced activity	44.43 (20.86)	−19.32	1.17	<0.001	35.99 (18.75)	−6.25	0.39	0.041	34.88 (18.60)	4.50	−0.74	<0.001	0.196	0.040
	25.11 (16.99)	−23.89 to −14.75	−2.39 to 4.72		29.74 (24.26)	−12.22 to −0.28	−4.69 to 5.47		39.38 (14.79)	2.47 to 6.53	−4.04 to 2.55		0.297	<0.001

Reduced motivation	40.28 (19.97)	−18.87	1.06	<0.001	28.22 (17.34)	−2.65	0.15	0.395	28.63 (18.17)	3.38	−0.56	<0.001	0.013	0.007
	21.41 (16.31)	−23.82 to −13.91	−2.35 to 4.46		25.57 (19.10)	−8.92 to 3.62	−4.12 to 4.42		32.00 (16.25)	1.57 to 5.18	−3.93 to 2.82		0.283	0.001

Mental fatigue	33.9 (23.3)	−15.34	0.78	<0.001	24.46 (20.30)	0.0	0.00	1.000	23.38 (16.41)	5.00	−0.75	<0.001	0.051	0.011
	19.9 (17.6)	−20.53 to −10.15	−3.08 to 4.63		24.46 (21.08)	−5.13 to 5.13	−4.85 to 4.85		28.38 (13.55)	2.94 to 7.06	−3.70 to 2.20		0.268	0.007

CFQ	31.52 (11.45)	−6.98	1.09	<0.001	26.59 (13.79)	−3.65	0.28	0.117	26.02 (9.50)	1.3	−0.58	0.005	0.172	0.054
	24.54 (10.40)	−8.76 to −5.21	−0.96 to 3.13		22.94 (9.79)	−8.26 to 0.97	−2.52 to 3.09		27.32 (7.36)	0.42 to 2.18	−2.25 to 1.08		0.476	0.121

Mood	2.52 (0.87)	1.10	−1.03	<0.001	2.94 (0.54)	0.34	−0.65	0.001	3.08 (0.53)	−0.02	0.05	0.766	0.031	<0.001
	3.62 (0.59)	0.49 to 0.94	−1.16 to −0.89		3.29 (0.46)	0.16 to 0.53	−0.77 to −0.54		3.06 (0.32)	−0.16 to 0.12	−0.04 to 0.13		0.033	<0.001

VAS	4.10 (2.74)	−3.38	1.95	<0.001	2.24 (2.02)	−0.21	0.64	0.006	2.06 (1.61)	−0.8	0.13	0.399	0.004	0.001
	0.71 (1.06)	−4.42 to −2.4	1.56 to 2.34		2.03 (1.77)	−0.35 to −0.06	0.19 to 1.08		1.98 (1.33)	−0.28 to 0.11	−0.16 to 0.42		0.001	<0.001

VAS: Visual Analog Scale (0–10 cm). CFQ: sum of overall cognitive failures;

G: geothermal, M: music, and C: control groups.

Baseline (B): state before treatment and after (A): state after treatment; ^a^95% CI: 95% confidence interval.
